# Neural mechanisms of deception in a social context: an fMRI replication study

**DOI:** 10.1038/s41598-020-67721-z

**Published:** 2020-07-01

**Authors:** Maya Zheltyakova, Maxim Kireev, Alexander Korotkov, Svyatoslav Medvedev

**Affiliations:** 0000 0001 2192 9124 grid.4886.2 N.P. Bechtereva Institute of the Human Brain, Russian Academy of Sciences, St. Petersburg, Russia

**Keywords:** Cognitive neuroscience, Social behaviour, Social neuroscience

## Abstract

Deception is a form of manipulation aimed at misleading another person by conveying false or truthful messages. Manipulative truthful statements could be considered as sophisticated deception and elicit an increased cognitive load. However, only one fMRI study reported its neural correlates. To provide independent evidence for sophisticated deception, we carried out an fMRI study replicating the experimental paradigm and Bayesian statistical approach utilized in that study. During the experiment, participants played a game against an opponent by sending deliberate deceptive or honest messages. Compared to truth-telling, deceptive intentions, regardless of how they were fulfilled, were associated with increased BOLD signals in the bilateral temporoparietal junction (TPJ), left precuneus, and right superior temporal sulcus (STS). The right TPJ participates in the attribution of mental states, acting in a social context, and moral behaviour. Moreover, the other revealed brain areas have been considered nodes in the theory of mind brain neural system. Therefore, the obtained results reflect an increased demand for socio‑cognitive processes associated with deceptive intentions. We replicated the original study showing the involvement of the right TPJ and expanded upon it by revealing the involvement of the left TPJ, left precuneus and right STS in actions with deceptive intentions.

## Introduction

All people occasionally engage in lying, which affects various areas of life, including politics, marketing, and personal relationships. Therefore, deception is an essential component of human behaviour. In the literature, deception is defined as a deliberate attempt to create a wrong belief in another person^1^. However, despite being the focus of numerous research articles^2–4^, the brain mechanisms of deception remain unclear.

The majority of researchers consider deception to be a more cognitively demanding task than telling the truth. According to the prevailing opinion, the reason for this is that telling false statements requires inhibition of a predominant truthful response^5–14^. However, deception is a complex phenomenon, uniting various aspects of cognition, including intentional, executive, social, linguistic, moral, and emotional aspects. Thus, its nature cannot be explained solely by the inhibition of truth without looking at other factors. Moreover, several neuroimaging studies have demonstrated the existence of processes other than inhibition of a predominant response as possibly associated with deception. In particular, deception is usually accompanied by increased activity in the frontal and parietal areas, which are associated with a combination of processes. All of these processes can explain higher cognitive load of deception: conflict monitoring, working memory, action selection, and inhibition^2,8,14–24^. Another reported neural correlate of deception is the error detection mechanism^25,26^. It activates in response to erroneous performance and is involved in the processing of deliberate deception because we do not believe our false statement^20,27^.

An additional aspect of deception was brought to attention due to the development of ecologically valid paradigms in the area of deception research. In these paradigms, unlike in previous studies, participants were making decisions on whether to lie or tell the truth to an interlocutor throughout the experiment^6,18,21,22,27–33^. The deliberate choice to deceive required evaluation of the current situation and possible outcomes and, in particular, inferring the mental state of the opponent to create a false belief. Therefore, several new paradigms have emphasized the importance of socio-cognitive mechanisms for successful deception^3,18,34^. From the socio-cognitive perspective, creating a false belief in another person is a form of manipulation. This challenges the predominant view of deception as only telling false statements and, therefore, suggests the re-consideration of existing definitions. Specifically, conveying part of the truth and not revealing all the relevant information is also a form of manipulation^1^. Even communicating a formally truthful statement sometimes fulfils the intention to create a false belief in others^35–37^. For example, in a situation where an interlocutor expects to be deceived, one can strategically choose to deceive by telling the truth. Some authors consider this action even more difficult to perform than simple deception and, therefore, have named it ‘sophisticated deception’^35^.

However, little attention has been given to examining neural correlates of sophisticated deception. The results of ERP studies have suggested that deceiving by truth is similar to simple deception in the sense that it is also associated with increased reaction times and higher demands on cognitive control and executive functions^28,29,31,32^. However, this form of falsification does not involve the process of inhibition of prepotent honest responses or activate the error detection mechanism mentioned above. Instead, it has been suggested that sophisticated deception is characterized by a higher load on socio-cognitive processes, which reflects its manipulative intention^35^. The reason is that to create a wrong belief in someone, it is necessary to infer the opinion of an interlocutor and keep in mind his or her mental state^28,32^.

This idea was confirmed in an fMRI study of deliberate deception, in which participants took part in the strategic game against the opponent by sending deceptive or truthful statements based on their own decisions^30^. The results revealed increased activity in the right temporoparietal junction (rTPJ) during communicating true or false statements with an intent to deceive compared to simply telling the truth. Additionally, higher activity in the bilateral TPJ was demonstrated when performing sophisticated deception than when deceiving by telling false statements. In the literature, the TPJ has been associated with social cognition and acting in a social context^38,39^ and has been specifically mentioned as one of the critical nodes of the brain neural system underlying theory of mind (i.e., the TOM network)^40–42^. The TOM network is considered to be associated with inferring thoughts and beliefs of other people^43–47^. Additionally, PPI analysis in the fMRI study, with a similar game design in which participants performed deceptive and manipulative honest claims according to free choice with the aim of defeating an opponent, revealed the involvement of this brain area^19^. During both manipulative actions, regardless of their truthfulness, the rTPJ was involved in interaction with the left middle frontal gyrus (lMFG), and no significant difference in functional interactions between the rTPJ and lMFG was observed when comparing deceptive and truthful conditions. Therefore, these data were used to support the idea that sophisticated deception (i.e., manipulative truth) and simple deception, which share manipulative aspects, are associated with local activity in the rTPJ and its distant functional interactions. These data reflect the involvement and the critical role of socio-cognitive processes in deception.

In a partial contradiction to these findings were the results of an fMRI study in which participants were presented with stories and decided whether to tell the truth or to lie in described scenarios^48^. Only deception in situations in which it was regarded as harmful elicited higher BOLD signals in the rTPJ than truth-telling. Therefore, local activity in the rTPJ may be related to the moral assessment of behaviour and self-monitoring. In line with this, another study has reported that transcranial direct-current stimulation of the rTPJ resulted in a significant decrease in the rate of successful deception^49^. A suggested explanation for these counterintuitive findings was that the neural enhancement of the rTPJ increased moral self-control and resulted in worse deceptive performance.

Therefore, it is still unclear whether the performance of simple deception and deception performed by truth are associated with a socio-cognitive process load and increased BOLD signals in the rTPJ. Current research suggests that the intention to deceive can be viewed as a form of manipulation; therefore, the socio-cognitive aspect is crucial for its performance. Based on this, higher local activity is expected to be observed in brain areas responsible for socio-cognitive processes while acting with the intention to deceive, regardless of how this manipulative intention is fulfilled. To examine this assumption, we conducted an fMRI experiment that replicated the same paradigm and statistical approach, namely, Bayesian inference, as reported in the study by Volz et al. (2015). During the experiment, participants played a strategic game against an opponent by sending deceptive or honest messages based on their free choice. Importantly, the present design allowed us to know the participant’s real intention and distinguish manipulative actions, simple and sophisticated deception, from non-manipulative plain truth-telling. Additionally, the control condition was plain truth-telling and was made in the same social context with the same free choice as in the manipulative conditions. Therefore, the current paper aims to stress the importance of the socio-cognitive aspects of deception, test the conclusions made by Volz and colleagues and expand the list of possible neural correlates of deception.

## Results

### Behavioural results

For the 23 participants included in the statistical analysis, the mean response time (RT) was equal to 2,565 ms (SD = 726 ms) for truth trials, 2,817 ms (SD = 872 ms) for simple deception trials, and 3,091 (SD = 1,190 ms) for sophisticated deception trials. Paired Student’s *t* tests showed that RTs for truth trials were significantly different from RTs for both simple (t = − 4.32, p = 0.0003) and sophisticated (t = − 4.01, p < 0.001) deception trials. At the same time, no significant difference in RTs between simple and sophisticated deception trials was revealed (t = − 1.39, p = 0.2). These results support that communicating truth with an intention to deceive (manipulative truth) differs from telling the plain truth but does not differ from deceiving by telling lies.

### Imaging results

#### Neural correlates of the intention to deceive (simple deception and sophisticated deception > truth)

**Figure 1:**
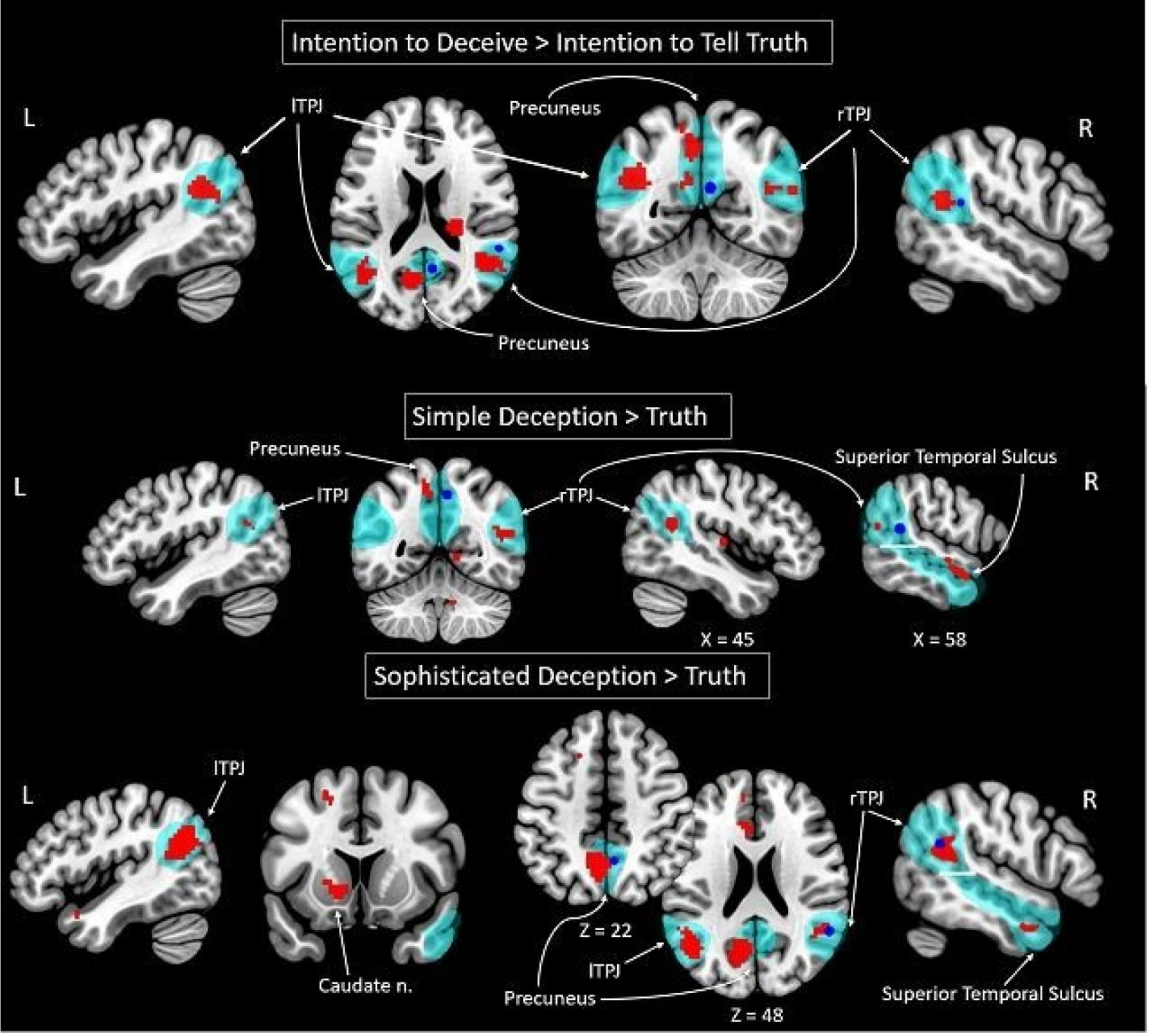
Clusters of the increased BOLD signal associated with simple deception and sophisticated deception claims according to Bayesian inference are shown in red. Clusters reported by Volz and colleagues are illustrated as spheres in blue (radius = 4 mm; center = maximum coordinates of clusters, reported for identical contrast). The theory of mind network clusters are shown as thresholded maps from the group analysis of the contrast false belief > false photograph^45^ in teal (downloaded at https://saxelab.mit.edu/use-our-theory-mind-group-maps). *L/R* left/right hemisphere, *g.* gyrus, *TPJ* temporoparietal junction.

To find neural correlates of the intention to deceive, we contrasted both types of deception trials (simple and sophisticated deception), combined together, against the truth trials. This comparison revealed an increase in the BOLD signal in the TPJ (bilaterally), left middle frontal gyrus (MFG), right anterior cingulate cortex (ACC), left precuneus, and bilateral cerebellum (see Fig. 1 and Table 1). The observed BOLD signal changes, located in the bilateral TPJ, spatially correspond to the activation of the rTPJ, as revealed in the study by Volz et al. (2015).

**Table 1:** BOLD signal changes associated with the intention to deceive, simple deception, and sophisticated deception (according to Bayesian inference for contrasts simple deception *and* sophisticated deception vs. truth, simple deception vs. truth, and sophisticated deception vs. truth).

Brain region	k	Log odds	Peak MNI coordinates		
			x	y	z
**Intention to deceive > truth**					
*R TPJ	98	8.90	42	− 49	20
*L TPJ	118	5.11	− 45	− 64	20
L MFG	29	4.80	− 12	− 13	65
R ACC	39	5.17	18	26	26
*L precuneus	208	7.15	− 9	− 52	50
R cerebellum	20	4.63	12	− 52	− 34
L cerebellum	105	6.74	0	− 43	− 16
L temporal lobe/white matter	143	7.04	33	− 46	8
R sub-lobar/extra-nuclear/white matter	48	6.77	30	− 10	20
L brainstem/midbrain	32	6.26	− 6	− 25	− 13
L brainstem/pons	29	5.79	− 6	− 37	− 34
R corpus callosum	27	5.37	9	14	20
R frontal lobe/white matter	20	4.42	27	26	8
**Simple deception > truth**					
*R TPJ	38	6.05	45	− 52	20
*L TPJ	10	3.39	− 45	− 61	23
L medial frontal g.	12	6.75	− 12	− 13	68
L SFG (BA 6)	11	4.64	− 21	8	59
R Insula	31	4.79	42	− 16	8
*L precuneus (BA 7)	35	5.26	− 12	− 52	53
*R MTG–STS TOM region (BA 21)	53	6.19	57	5	− 16
L MTG	108	7.47	− 51	− 22	− 7
R STG	11	4.89	39	8	− 31
L middle occipital g. (BA 18)	23	6.21	− 27	− 94	2
L putamen	18	6.09	− 27	− 1	8
L caudate nucleus	11	4.76	− 18	2	20
R cerebellum	78	8.49	3	− 43	− 13
	18	5.28	12	− 52	− 34
L cerebellum	53	7.78	− 15	− 34	− 22
L brainstem/midbrain	38	7.31	− 6	− 25	− 13
R corpus callosum	10	6.52	6	20	20
R sub-lobar/extra-nuclear/white matter	21	6.50	30	− 46	8
	15	4.13	6	− 1	− 1
**Sophisticated deception > truth**					
*R TPJ	84	8.25	45	− 49	20
*L TPJ	185	6.90	− 45	− 61	23
L MFG	26	6.10	− 27	29	32
R SFG	20	4.94	21	20	38
L SFG	25	4.93	− 18	14	53
R Insula (BA 47)	30	5.64	30	20	− 7
L ACC	51	6.29	− 12	41	− 1
	53	6.28	− 12	29	32
*L precuneus	350	8.84	− 6	− 55	47
L MTG (BA 21)	45	7.40	− 54	5	− 25
*R ITG–STS TOM region (BA 20)	51	6.64	48	− 1	− 31
L caudate nucleus/caudate head	21	7.14	− 12	17	− 4
R cerebellum	30	7.08	9	− 49	− 31
	295	9.26	9	− 43	− 7
R sub-lobar/white matter/anterior commissure	76	8.16	3	2	− 1

*L/R* left/right hemisphere, *k* cluster size in voxels, *g.* gyrus, *BA* approximate Brodmann’s area, *TPJ* temporoparietal junction, *MFG* middle frontal gyrus, *SFG* superior frontal gyrus, *ACC* anterior cingulate cortex, *STG* superior temporal gyrus, *MTG* middle temporal gyrus, *STS* superior temporal sulcus, *TOM* theory of mind, *ITG* inferior temporal gyrus; *clusters with coordinates of maximums lying within TOM-related brain regions according to thresholded maps^45^ (downloaded at https://saxelab.mit.edu/use-our-theory-mind-group-maps).

#### Neural correlates of lying (simple deception > truth)

Performing simple deception (deceiving by telling false statement), compared to telling the truth, corresponded to increased activity in the TPJ (bilaterally), left medial frontal gyrus, left superior frontal gyrus (SFG), right insula, left precuneus, left middle temporal gyrus (MTG), right superior temporal sulcus (STS; peak located in the right MTG), right superior temporal gyrus (STG), left occipital cortex, left putamen, left caudate nucleus, and bilateral cerebellum (see Fig. 1 and Table 1). The revealed cluster in the bilateral TPJ partially replicated the activity in the rTPJ, detected by Volz et al. (2015), and supports the relevance of treating the communication of false statements as a form of manipulation.

#### Neural correlates of sophisticated deception (sophisticated deception > truth)

Contrasting sophisticated deception trials with truth trials allowed us to test for the difference in brain activity between the truth that is told with and without an intention to deceive. For sophisticated deception, greater activity in the bilateral TPJ, left MFG, bilateral SFG, left ACC, right insula, left precuneus, left MTG, right STS (peak located in the right inferior temporal gyrus), left caudate nucleus, and right cerebellum was observed (see Fig. 1 and Table 1).

These findings confirmed that performing sophisticated deception is not the same as telling the truth; otherwise, no significant difference between these two types of trials would have been revealed. In addition, in comparing sophisticated deception with truth, we found activity similar to that discovered in the contrast between simple deception and truth. This supports the assumption that manipulative truth can, in fact, be called sophisticated deception. This is consistent with the conclusions of Volz et al. (2015); however, activity in the bilateral TPJ, instead of the rTPJ, was detected.

#### Neural correlates delineating two forms of deception (simple deception vs. sophisticated deception)

**Figure 2:**
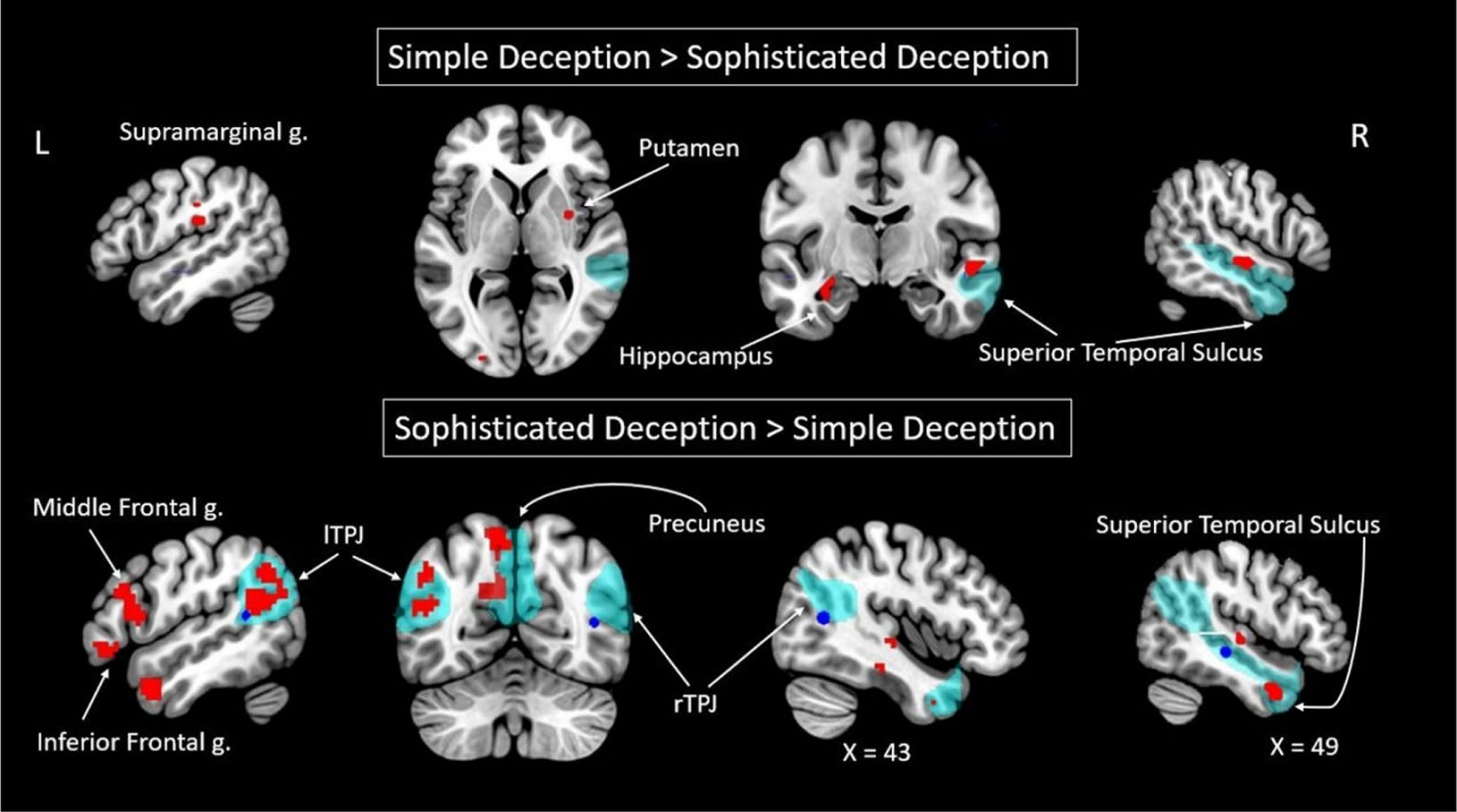
Clusters with significant differences in BOLD signals that delineate two forms of deception–simple deception and sophisticated deception–according to Bayesian inference are shown in red. Clusters reported by Volz and colleagues are illustrated as blue spheres (radius = 4 mm, center = maximum coordinates of reported clusters for identical contrast). The theory of mind network clusters is shown as thresholded maps from the group analysis of the contrast false belief > false photograph^45^ in teal (downloaded at https://saxelab.mit.edu/use-our-theory-mind-group-maps). *L/R* left/right hemisphere, *g.* gyrus, *TPJ* temporoparietal junction.

To distinguish neural correlates of these two kinds of manipulative actions, performed with the intention to deceive, we contrasted simple deception and sophisticated deception conditions. The manipulative intention, fulfilled by telling a false statement, corresponded to the increased BOLD signal in the right SFG, right postcentral gyrus, bilateral precentral gyrus, left supramarginal gyrus, right STS (peak located in the right MTG), left hippocampus, right putamen, and left occipital cortex (see Fig. 2 and Table 2). Truth, performed with the manipulative intention to deceive, shows increased BOLD signals in the left TPJ, left inferior frontal gyrus, bilateral MFG, bilateral SFG, right insula, left precuneus, right STS (peak located in the right MTG), left MTG, right STG, and right parahippocampal gyrus (see Fig. 2 and Table 2). These results replicated only the activity in the left TPJ, while Volz et al. (2015) reported activity in the bilateral TPJ during sophisticated deception compared to simple deception.

**Table 2:** BOLD signal changes delineating two forms of deception (according to Bayesian inference for the contrasts simple deception trials vs. sophisticated deception trials).

Brain region	k	Log odds	Peak MNI coordinates		
			x	y	z
**Simple deception > sophisticated deception**					
R SFG	23	5.79	15	41	53
R precentral g.	38	7.54	18	− 19	71
L precentral g.	38	7.64	− 9	− 19	68
R postcentral g.	33	5.81	21	− 37	65
L IPL/supramarginal g.	30	4.86	− 51	− 25	23
*R MTG–STS TOM region	29	6.86	54	− 13	− 7
L middle occipital g. (BA 18)	15	4.17	− 27	− 94	2
R putamen	20	4.03	30	− 1	5
L hippocampus	15	3.88	− 33	− 10	− 19
**Sophisticated deception > simple deception**					
*L TPJ	263	8.41	− 60	− 46	26
L IFG/pars orbitalis	45	5.88	− 48	35	− 7
R MFG (BA 9)	27	4.08	33	23	41
L MFG	160	5.47	− 39	38	26
R SFG	62	11.49	18	11	53
L SFG	56	7.33	− 15	20	56
	93	5.41	− 21	50	17
R insula (BA 47)	24	4.93	30	20	− 7
*L precuneus	86	5.89	− 15	− 76	26
	143	5.39	− 9	− 49	41
R parahippocampal g.	84	6.09	27	− 22	− 22
*R MTG (BA 21)–STS TOM region	44	8.58	48	2	− 31
L MTG (BA 38)	368	8.92	− 48	5	− 25
R STG (BA 22)	16	5.42	48	− 19	2
R brainstem/midbrain	163	8.59	3	− 22	− 16
L brainstem/midbrain	132	6.70	− 6	− 28	− 1
R sub-lobar/lateral ventricle/cerebro-spinal fluid	17	5.31	15	14	20
R temporal lobe/sub-gyral/white matter	116	6.11	24	− 46	− 1
R corpus callosum	44	7.88	9	32	5

*L/R* left/right hemisphere, *k* cluster size in voxels, *g.* gyrus, *BA* approximate Brodmann’s area, *SFG* superior frontal gyrus, *IPL* inferior parietal lobule, *MTG* middle temporal gyrus, *STS* superior temporal sulcus, *TOM* theory of mind, *TPJ* temporoparietal junction, *IFG* inferior frontal gyrus, *MFG* middle frontal gyrus, *STG* superior temporal gyrus; *clusters with coordinates of maximums lying within TOM-related brain regions according to thresholded maps^45^ (downloaded at https://saxelab.mit.edu/use-our-theory-mind-group-maps).

## Discussion

The main finding of the current replication study is that both simple and sophisticated deception (i.e., honest manipulative actions), compared to non-manipulative truth, were associated with increases in BOLD signals in several brain regions, including the TPJ region in both brain hemispheres. This result replicates the findings of Volz and colleagues regarding the involvement of the rTPJ in actions with deceptive intentions and thus confirms the critical role played by socio-cognitive processes in deception. Our findings also expand upon previous experimental data by demonstrating the increase in the BOLD signals in the left TPJ that was associated with the intention to deceive (simple and sophisticated deception).

However, it is important to mention that not all results of Volz and colleagues were replicated in our study. The reason for this may be in the different software packages used for fMRI data preprocessing and statistical analysis: the software package LIPSIA (Leipzig Image Processing and Statistical Inference Algorithms) version 2.2^50^ in the original study and the SPM12 software package in the current paper.

We also found that both simple deception and sophisticated deception statements, separately compared to truth statements, elicited higher RTs and higher BOLD signals in the bilateral TPJ, left precuneus and right STS. Consequently, these results support the opinion that a higher cognitive load of deception comes from deceptive intentions, regardless of the way they are fulfilled^28,29,31,32^. Evidence from the current study and Volz et al. (2015) is more precise than other studies in the literature because participants’ intentions were known and control truth statements, which involved the same free choice and social context, were made during the same session. Therefore, the observed differences in RTs and the neural activity of the correlates of socio-cognitive processes could be attributed exactly to the manipulative intentions.

Moreover, when directly comparing the two types of manipulative conditions, we observed that sophisticated deception elicited higher BOLD signals in areas of the TOM network, namely, the lTPJ and left precuneus, among other regions. Similar differences in activity were also found when comparing actions with manipulative intentions to truth-telling. Therefore, these results may indicate that sophisticated deception differs from simple deception by the degree of recruitment of socio-cognitive processes. This is also in line with the results of Volz and colleagues, who stated that sophisticated deception, compared to simply telling a lie, involved an increased load on TOM-related processes.

In accordance with data in the literature, both the right and left TPJ are included in the brain network of the so-called ‘mentalizing’ or ‘TOM’ brain system^40,43–47,51,52^. It was hypothesized that the TOM system is recruited by acting in settings implying social interactions that require attribution of mental states such as beliefs or desires to other people, understanding the intention of observed actions, and taking someone else’s perspective. These processes are essential for integrating social context and social decision-making^38,39^. Manipulation through creating incorrect beliefs is a representation of behaviour in a social context. From this, we infer that the increased BOLD signals in the bilateral TPJ reflect involvement of socio-cognitive processes in deception. It was previously found that the TOM system was relevant for performing deception. Only children with TOM can tell a lie^53,54^. In neuroimaging studies, the bilateral TPJ was reported to be associated with the judgement of deception^55,56^. However, only the rTPJ was demonstrated to increase activity during deception^3,19,30,48,49^. Therefore, the current results confirm the involvement of socio-cognitive processes in deception and expand the existing fMRI data, demonstrating that BOLD signal increases in both the right and left TPJ during the execution of deception.

Importantly, making both simple deception and sophisticated deception statements was associated with increased BOLD signals not only in the bilateral TPJ but also in the left precuneus and right STS. Volz et al. (2015) also reported altered activity in the right precuneus in the same comparison. These data provide additional evidence that the observed neural activity reflected the involvement of socio-cognitive processes. The left precuneus and right STS are also nodes in the brain network of the TOM system^44–47^. Additionally, the precuneus was reported to be involved in observing social interaction and cooperation, social emotion perception, and understanding body language^57–59^. Observed together, the increased local activity in the bilateral TPJ, left precuneus, and right STS confirms that these are socio-cognitive processes that are recruited to perform actions with manipulative intentions.

In line with this conclusion is the observation that neural correlates of deception depend on whether the experimental task involves a social context or not. For example, a meta-analysis showed that compared to truth-telling, deception elicited increased BOLD signals in the rTPJ only in studies that provided an interactive social context^3^. Similarly, the performance of deception by children aged 8–9 years in a social context, but not in less social context conditions, was associated with the BOLD signals in the right precuneus^60^. Additionally, in a study examining deliberate deception in which the participants played a game with a computer algorithm, no significant difference was revealed in the local activity of areas related to TOM system when comparing the performance of manipulative actions (deception and manipulative truth) and the performance of the control task without manipulation^20^. Interactions with computers result in subdued activity in the TOM system compared to that observed during interactions with humans^61–66^. Taking this into account, we assume that the activity of the TPJ, precuneus, and STS during deception can also be modulated by the human-likeness of the opponent. Therefore, the differences in the BOLD signals observed in the current study were driven by a socio-cognitive aspect of deception provided by the game with human opponents.

Given the role of the socio-cognitive aspects of deception, the brain mechanisms can be further studied in clinical research and criminology. It has already been shown that clinical disorders, including schizophrenia, borderline personality, and autism spectrum disorders, are linked to altered activity and connectivity of TOM-related regions in the human brain^67,68^. Additionally, individuals with psychopathic traits are believed to have dysfunction in TOM processes^69,70^, which can lead to distorted attitudes towards deception^71–73^.

## Conclusion

In conclusion, this fMRI replication study points out that socio-cognitive processes are an essential part of deceptive behaviour. We characterized deception as a form of manipulation that is characterized by the intention to create a wrong belief in an interlocutor. We demonstrated that deceptive intentions, regardless of whether their goal was achieved, enacted by telling false (simple deception) or honest manipulative (sophisticated deception) statements, compared to acting without an intention to deceive (truth), were associated with increased BOLD signals in a number of areas, including the bilateral TPJ, left precuneus and right STS. These areas are associated with acting in social settings and social decision-making and are usually considered nodes in the TOM brain system, which is associated with the process of attributing mental states to others. The obtained results not only replicate but also expand the results of Volz et al. (2015); in addition to the involvement of the rTPJ and right precuneus, the involvement of the lTPJ, left precuneus and right STS in actions with deceptive intentions was revealed.

## Materials and methods

### Participants

Thirty-three (19 male, 14 female) right-handed volunteers, all native Russian speakers, without any history of neurological or psychiatric disorders or current medication intake participated in the fMRI study for a monetary reward (1,000 rubles). Participants could also win an additional 300 rubles during the game. The total amount of reward was defined at the end of the experiment when one trial was randomly chosen, and volunteers were paid according to the number of game points won in that trial. Handedness was assessed with the Edinburgh Handedness Inventory^74^. All subjects gave their written informed consent before the study. All procedures were in accordance with the declaration of Helsinki and were approved by the Ethics Committee of the N.P. Bechtereva Institute of the Human Brain, St. Petersburg, Russia. Ten participants were excluded from the statistical analysis because of too few trials of a particular kind (6 participants made either none or too few sophisticated deception claims, and 4 participants made too few simple deception claims).

### Stimuli and procedure

The experimental design used in this study was described in the paper by Volz et al. (2015). For the current study, we translated the participant instructions and stimuli to the Russian language.

In the experiment, the participants played a “sender-receiver” game while being scanned. Before the beginning of the game, they were instructed that they would play against a real human opponent to create an interactive social context. However, this information was untrue, and a computer randomly generated all actions of their opponent.

**Figure 3:**
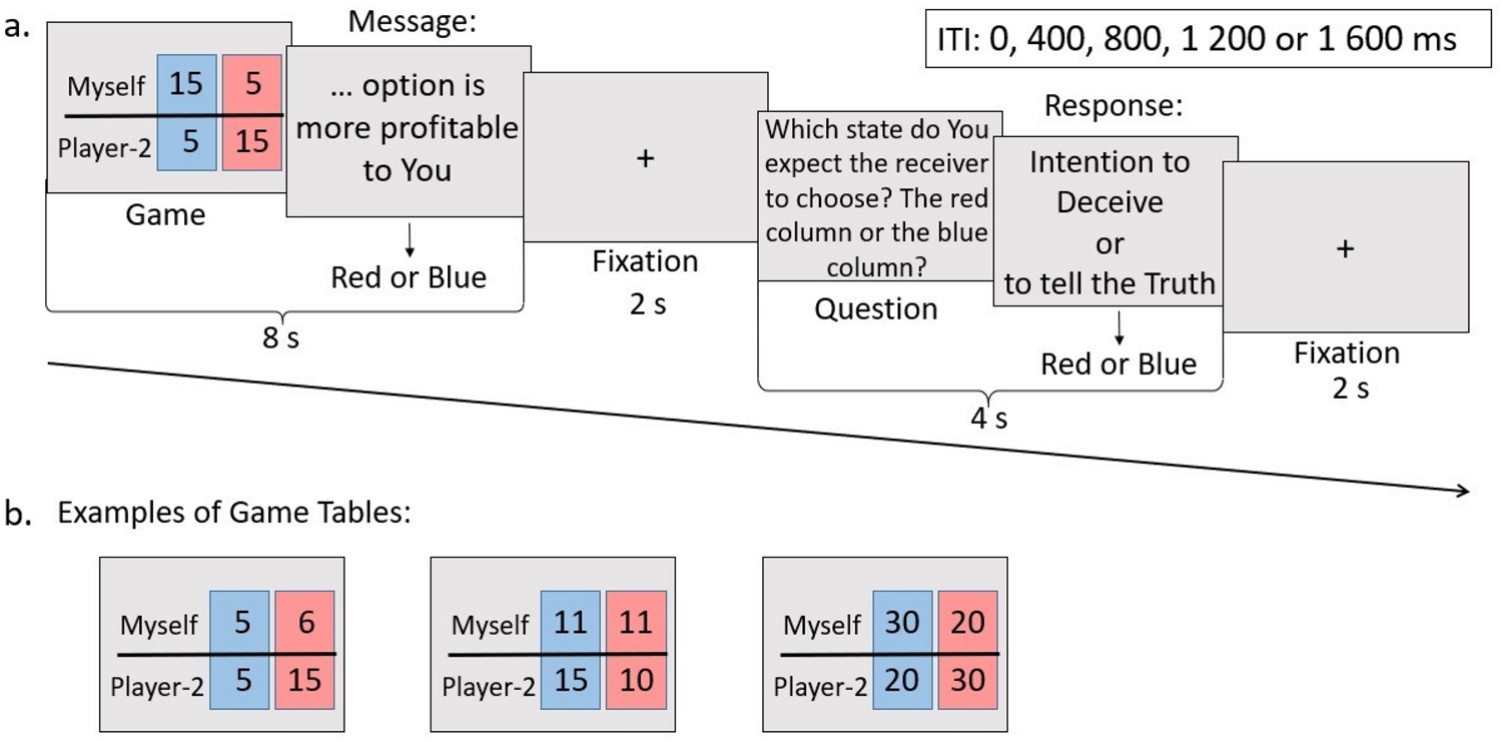
(**a**) Structure of the “sender-receiver” game. In each trial, the participants saw the possible points scored for choosing red and blue options for both players. Pressing a button that corresponded to either the red or blue option, they sent a truth or deception message to their opponents (“Red/Blue option is more profitable to You”). Next, the participants answered a question about their expectations by pressing the red or blue button. These answers revealed their intentions and separated the *truth* and *sophisticated deception* trials: in both cases, the participants sent a true message, but in *truth*, they expected the opponents to believe them, while in *sophisticated deception*, they expected opponents to not believe and choose the opposite option. Next, a new trial began (no feedback was shown until all 90 trials were finished). (**b**) Examples of game tables that the participants saw at the beginning of each trial. They show the distributions of points for both participants corresponding to the choice of the red or blue option in that trial.

During the game, a participant in the scanner saw two possible options of monetary consequences, both for oneself and for the opponent, with one option associated with blue and another associated with red (see Fig. 3). The participant was instructed to send information to a second player that said either “Blue option is more profitable to You” or “Red option is more profitable to You” by pressing the corresponding left or right button on the controller. In each trial, one of the messages was true, and another was false. After that, the participant considered which option he or she expected the opponent to choose and responded to the question: “Which state do you expect the receiver to choose? The red column or the blue column?”. Next, a new trial started. The participants believed that their opponent received the message and chose the blue or red variant, which determined final payoffs for both players. However, to prevent learning and adjusting strategy, the person did not see the results of choices after each trial.

The game consisted of 90 trials divided into two sessions. Relative monetary gains for the two players varied between trials. In 45 of the trials, two options were available: one was more profitable for the participant and less profitable for the opponent, and another had the opposite outcomes. In 27 trials, the participant earned the same amount of money in both cases, but the monetary payoff for the opponent varied and could be higher, lower or the same as that of the participant. In 18 trials, one option contained higher profits for both players. The order of trial presentation was randomized. The complete list of all distributions of monetary payoffs, viewed by the participants, is provided in Supplementary Materials (see Table A–Table C).

It is important to note that the participant believed that the opponent did not know the distribution of gains between the two options, even at the end of the trial. Therefore, the participant could send either truth or deception claims, according to one’s own decision. Since only the opponent could choose final payoffs, manipulating this choice by sending messages was the only opportunity to influence the results of the trial. After all 90 trials were played, one trial was randomly chosen to determine an additional payment to the participant for taking part in the experiment.

**Figure 4:**
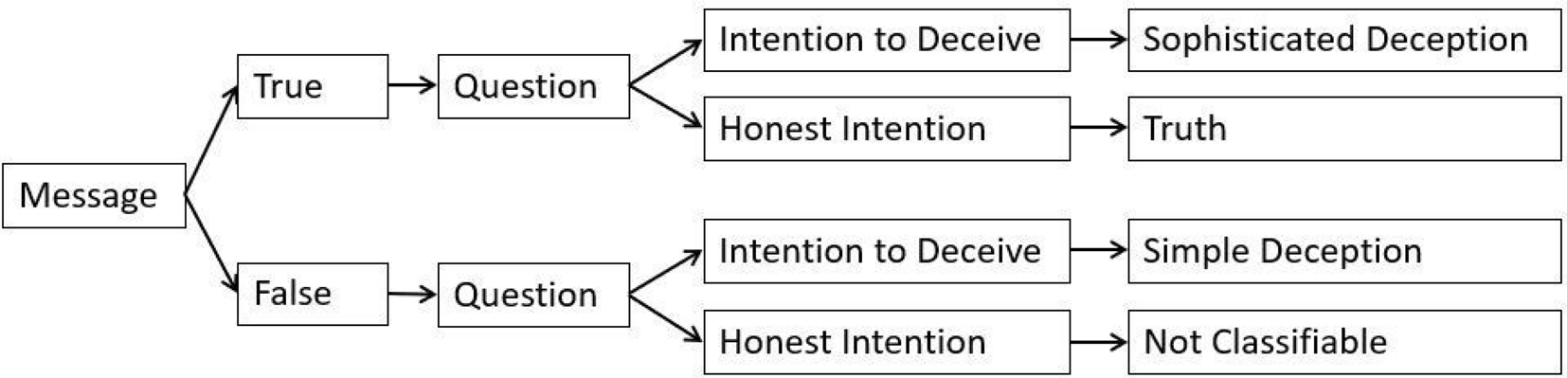
Trial classification. “Message” refers to the information sent to the opponent by the first button press: “Red/Blue option is more profitable to You”. “Question” refers to the answer to the second question: “Which state do you expect the receiver to choose? The red column or the blue column?”.

The claim was classified as *truth* when the participant sent an honest message and expected the opponent to choose the same option as was stated in the message (see Fig. 4). *Simple deception* was defined as a trial in which a false message was sent, and it was expected to be accepted by the opponent. Choices in which the participant's message was truthful but the expectation was that the opponent would not believe in the message were defined as *sophisticated deception*. The participants could also send deceptive messages and expect their opponents to choose a truthful statement instead. This kind of claim was not attributed to any classified trial category and was called *not classifiable*. Trials in which the participants did not send a message, did not answer the question or did not do either of those were excluded and considered as a condition of no interest in the statistical analysis.

All trials lasted for 16 s. The table with possible monetary payoffs was present on the screen for 8 s, during which participants could respond by pressing the button on the controller. Then, the fixation cross appeared on the screen for 2 s. After that, the question about the participant’s expectations was presented for 4 s, followed by the fixation cross for 2 s. The interval between trials was randomly chosen from the following: 0, 400, 800, 1,200, and 1,600 ms.

The presentation of stimuli, recording participant responses, and synchronization of them with functional image acquisition were implemented by NordicNeuroLab and E-Prime software (version 1.1, Psychology Software Tools Inc., Pittsburgh, PA, USA).

### fMRI image acquisition procedure

Magnetic resonance imaging was performed using a 3 T Philips Achieva (Philips Medical Systems, Best, The Netherlands). Structural images were acquired using a T1-weighted pulse sequence (T1W-3D-FFE; repetition time (TR) = 2.5 ms; echo time (TE) = 3.1 ms; 30° flip angle), measuring 130 axial slices (field of view (FOV) = 240 × 240 mm; 256 × 256 scan matrix) of 0.94 mm thickness. Functional images were obtained using an echo planar imaging (EPI) sequence (TE = 35 ms; 90° flip angle; FOV = 208 × 208 mm; 128 × 128 scan matrix). Thirty-two continuous 3.5-mm-thick axial slices (voxel size = 3 × 3 × 3.5 mm), covering the entire cerebrum and most of the cerebellum, were oriented with respect to the structural image. The images were acquired using a TR of 2,000 ms.

### Image processing and statistical analysis

Pre-processing and statistical analysis of the fMRI data was performed with Statistical Parametric Mapping 12 software (SPM12, https://www.fil.ion.ucl.ac.uk/spm) running in MATLAB (Mathworks Inc., Natick, MA, USA). The pre-processing procedure for each subject included spatial image realignment to the first functional image. After that, the slice-time correction was applied to adjust for differences in time of acquisition for each slice. Finally, functional images were normalized to a standard stereotactic MNI template (Montreal Neurological Institute) and smoothed (8-mm full-width at half-maximum). The cervical MRI-compatible collar “Philadelphia” was used during the data acquisition to prevent participant head motions.

At the first stage of the fMRI analysis, general linear models (GLMs) for each participant were generated. Regressors of interest corresponded to the following experimental trials: *truth*, *simple deception*, and *sophisticated deception*. Additionally, regressors of no interest for *not classifiable* trials, trials with mistakes as well as for *question* stimuli were used to regress out BOLD signal changes, connected to making non-relevant, not classifiable claims and giving responses to questions. *Truth*, *simple deception*, *sophisticated deception*, and *non-classifiable* events were modelled with the onset at the beginning of a new trial and a duration equal to the time taken to press the button in each trial (message reaction time). *Question* events were modelled with the onset at the beginning of the question presentation (“Which state do you expect the receiver to choose? The red column or the blue column?”) and duration equal to the amount of time before participants pressed the button to answer the question (question reaction time). Each regressor was convolved with a canonical haemodynamic response function (HRF), and a temporal high-pass filter (cut-off: 128 s) was applied. Aside from the five previously mentioned regressors, GLMs also included six parameters of head movement produced at the realignment stage. For each participant, beta values of regression coefficients were estimated, and t-contrasts between these values for regressors of interest and baseline BOLD signals were calculated (*truth* > *baseline*, *simple deception* > *baseline*, *sophisticated deception* > *baseline*).

In the second stage, the resulting contrasts were transferred to the group level of statistical analysis, during which a flexible factorial analysis with one factor (condition) and three levels (truth, simple deception, sophisticated deception) was performed. For the results’ estimation and making inferences, the Bayesian inference (as is implemented in the SPM12) was used. The chosen statistical method has several advantages over the standard statistical approach. Standard or, so-called, frequentist statistics use zero hypothesis significance testing, which leads to the multiple comparisons problem, while the necessary correction for this problem leads to type II errors (existing differences are not detected). The alternative method, based on Bayesian statistics, estimates the presence or absence of the effect of interest in a group of participants based on the calculation of the posterior probability for obtaining the effect. It is calculated by correcting the prior probability distribution and likelihood given the obtained data. In the context of the present study, the posterior probability refers to the probability of the difference between conditions (i.e., the contrasts obtained during the 1st level analysis) to be larger than zero. As no significance test is performed, no correction for multiple comparisons is required. Additionally, no effect size threshold is necessary to apply in the Bayesian approach^75^. In the present study, a voxelwise analysis for pairs and groups of contrasts was performed using a zero effect size threshold (same as applied in the paper by Volz et al. (2015)) and a PPM threshold defined as log-odds threshold > 3. Xjview Toolbox (https://www.alivelearn.net/xjview/) was used to identify the anatomical location. Clusters lying within the TOM system were distinguished and labelled according to thresholded maps of seven TOM regions: the rTPJ and lTPJ, the precuneus, the dorsal, middle and ventral components of the medial prefrontal cortex, and the right STS^45^ (downloaded at https://saxelab.mit.edu/use-our-theory-mind-group-maps).

## Supplementary information

Supplementary file1
