# Supplementary material for: Neural mechanisms of deception in a social context: an fMRI replication study
Source: Sci Rep. 2020 Jul 1;10:10713. doi: 10.1038/s41598-020-67721-z (PMC7329834; doi:10.1038/s41598-020-67721-z)
Supplement: Supplementary file 1 — Supplementary file1 [file 41598_2020_67721_MOESM1_ESM.docx]

**Neural mechanisms of deception in social context: an fMRI replication study**

**Maya Zheltyakova, Maxim Kireev, Alexander Korotkov, Svyatoslav Medvedev**

**Full list of game tables, employed in the experiment.** Sender Red: payoff for the participant when Red option is chosen, sender Blue: payoff for the participant when Blue option is chosen, receiver Red: payoff for the opponent when Red option is chosen, receiver Blue: payoff for the opponent when Blue option is chosen (see also Fig. 1 in the Article file).

**Table A.**

List of game tables, in which one option was more profitable for the participant and less profitable for the opponent and another option had the opposite outcomes (n = 45).

| Sender Red | Receiver Red | Sender Blue | Receiver Blue |
| --- | --- | --- | --- |
| 1 | 0 | 0 | 1 |
| 5 | 6 | 6 | 5 |
| 11 | 10 | 10 | 11 |
| 16 | 15 | 15 | 16 |
| 20 | 21 | 21 | 20 |
| 5 | 0 | 0 | 5 |
| 5 | 10 | 10 | 5 |
| 15 | 10 | 10 | 15 |
| 20 | 15 | 15 | 20 |
| 20 | 25 | 25 | 20 |
| 0 | 10 | 10 | 0 |
| 15 | 5 | 5 | 15 |
| 10 | 20 | 20 | 10 |
| 25 | 15 | 15 | 25 |
| 20 | 30 | 30 | 20 |
| 0 | 5 | 1 | 0 |
| 5 | 10 | 6 | 5 |
| 10 | 15 | 11 | 10 |
| 16 | 15 | 15 | 20 |
| 20 | 25 | 21 | 20 |
| 1 | 0 | 0 | 10 |
| 5 | 15 | 6 | 5 |
| 10 | 20 | 11 | 10 |
| 15 | 25 | 16 | 15 |
| 21 | 20 | 20 | 30 |
| 5 | 0 | 0 | 10 |
| 10 | 5 | 5 | 15 |
| 15 | 10 | 10 | 20 |
| 20 | 15 | 15 | 25 |
| 20 | 30 | 25 | 20 |
| 0 | 1 | 5 | 0 |
| 5 | 6 | 10 | 5 |
| 15 | 10 | 10 | 11 |
| 20 | 15 | 15 | 16 |
| 20 | 21 | 25 | 20 |
| 0 | 1 | 10 | 0 |
| 15 | 5 | 5 | 6 |
| 10 | 11 | 20 | 10 |
| 15 | 16 | 25 | 15 |
| 30 | 20 | 20 | 21 |
| 10 | 0 | 0 | 5 |
| 15 | 5 | 5 | 10 |
| 10 | 15 | 20 | 10 |
| 15 | 20 | 25 | 15 |
| 30 | 20 | 20 | 25 |

**Table B.**

List of game tables, in which participant earned the same amount of money in both options, but the monetary payoff for the opponent varied and could be higher, lower or the same as the one of the participant (n = 27).

| Sender Red | Receiver Red | Sender Blue | Receiver Blue |
| --- | --- | --- | --- |
| 1 | 0 | 1 | 1 |
| 6 | 5 | 6 | 6 |
| 11 | 10 | 11 | 11 |
| 1 | 5 | 1 | 0 |
| 6 | 10 | 6 | 5 |
| 11 | 15 | 11 | 10 |
| 1 | 0 | 1 | 10 |
| 6 | 15 | 6 | 5 |
| 11 | 20 | 11 | 10 |
| 5 | 0 | 5 | 5 |
| 10 | 5 | 10 | 10 |
| 15 | 10 | 15 | 15 |
| 5 | 10 | 5 | 0 |
| 10 | 15 | 10 | 5 |
| 15 | 20 | 15 | 10 |
| 5 | 0 | 5 | 15 |
| 10 | 5 | 10 | 20 |
| 15 | 10 | 15 | 25 |
| 10 | 10 | 10 | 0 |
| 15 | 15 | 15 | 5 |
| 20 | 20 | 20 | 10 |
| 10 | 0 | 10 | 15 |
| 15 | 5 | 15 | 20 |
| 20 | 25 | 20 | 10 |
| 10 | 0 | 10 | 20 |
| 15 | 5 | 15 | 25 |
| 20 | 30 | 20 | 10 |

**Table C.**

List of game tables, in which one option contained higher profits for both players (n = 18).

| Sender Red | Receiver Red | Sender Blue | Receiver Blue |
| --- | --- | --- | --- |
| 1 | 1 | 0 | 0 |
| 0 | 0 | 1 | 5 |
| 1 | 10 | 0 | 0 |
| 5 | 5 | 6 | 6 |
| 6 | 10 | 5 | 5 |
| 5 | 5 | 6 | 15 |
| 10 | 10 | 11 | 11 |
| 11 | 15 | 10 | 10 |
| 10 | 10 | 11 | 20 |
| 5 | 1 | 0 | 0 |
| 0 | 0 | 5 | 5 |
| 5 | 10 | 0 | 0 |
| 5 | 5 | 10 | 6 |
| 10 | 10 | 5 | 5 |
| 5 | 5 | 10 | 15 |
| 10 | 10 | 15 | 11 |
| 15 | 15 | 10 | 10 |
| 15 | 20 | 10 | 10 |
